# The Association Between Serum *α*‐Tocopherol and Pathogenesis of Multiple Sclerosis: A Systematic Review and Meta‐Analysis of Case‐Control Studies

**DOI:** 10.1155/bmri/2590873

**Published:** 2026-07-08

**Authors:** Hamid Abbasi, Behnaz Ahmadi, Navid Sherafati, Reza Ansarian, Seyedyashar Pourebrahimian Leilabadi, Sina Hamzehzadeh, Reyhane Dehzadeh, Mahnaz Talebi, Sarvin Sanaie, Leila Nikniaz, Amirreza Naseri

**Affiliations:** ^1^ Student Research Committee, Faculty of Nutrition and Food Technology, Shahid Beheshti University of Medical Sciences, Tehran, Iran, sbmu.ac.ir; ^2^ Neurosciences Research Center (NSRC), Tabriz University of Medical Sciences, Tabriz, Iran, tbzmed.ac.ir; ^3^ Department of Nutrition, School of Health and Nutrition, Yasuj University of Medical Sciences, Yasuj, Iran, yums.ac.ir; ^4^ Student Research Committee, Tabriz University of Medical Sciences, Tabriz, Iran, tbzmed.ac.ir; ^5^ Research Center for Evidence-Based Medicine, Iranian EBM Centre: A Joanna Briggs Institute (JBI) Center of Excellence, Tabriz University of Medical Sciences, Tabriz, Iran, tbzmed.ac.ir; ^6^ Resuscitation and Critical Care Medicine Research Center, Tabriz University of Medical Sciences, Tabriz, Iran, tbzmed.ac.ir; ^7^ Tabriz Health Services Management Research Center, Tabriz University of Medical Sciences, Tabriz, Iran, tbzmed.ac.ir; ^8^ Research Center for Integrative Medicine in Aging, Aging Research Institute, Tabriz University of Medical Sciences, Tabriz, Iran, tbzmed.ac.ir; ^9^ Tabriz USERN Office, Universal Scientific Education and Research Network (USERN), Tabriz, Iran, usern.tums.ac.ir

**Keywords:** *α*-tocopherol, alpha-tocopherol, meta-analysis, multiple sclerosis, vitamin E

## Abstract

**Background:**

Oxidative stress is a key pathological mechanism of multiple sclerosis (MS) and *α*‐tocopherol is recognized as a primary chain‐breaking antioxidant. This study is aimed at investigating the relationship between serum *α*‐tocopherol and the pathogenesis of MS

**Methods:**

This systematic review was conducted in accordance with the Preferred Reporting Items for Systematic Reviews and Meta‐Analyses (PRISMA) statement and the Joanna Briggs Institute (JBI) manual for evidence synthesis. Scopus, PubMed, Web of Science, and Embase were searched in March 2025. The risk of bias was evaluated using the critical appraisal tool developed by JBI. All statistical analyses were performed with comprehensive meta‐analysis software (CMA3).

**Results:**

Out of 506 records evaluated during the title/abstract screening phase, five case‐control studies were included. Studies encompassed a total of 389 participants, comprising 211 individuals diagnosed with MS and 178 healthy controls. The levels of *α*‐tocopherol were found to be lower in MS patients compared to the healthy group (standardized mean difference [SMD] = −1.17; 95% CI: −2.02, −0.31; *p* = 0.007, *I* ^2^ = 91.5*%*, *p* < 0.001; predictive values: −3.13, 0.79). Although subgroup analyses were performed based on sample size, sex, and phase of the diseases, the source of heterogeneity was not found.

**Discussion:**

Current evidence supports the possible association of serum *α*‐tocopherol and MS pathogenesis; however, considering the restricted number of included studies (five papers), the retrospective nature of the studies, and substantial heterogeneity in the conducted quantitative synthesis, multicenter and large‐scale prospective studies are recommended.

## 1. Introduction

Multiple sclerosis (MS) is a predominantly immune‐mediated disease that affects the central nervous system (CNS) [1]. The worldwide prevalence of MS is an upward trend that affects nearly 2.8 million individuals [2]. Prevalent neurological symptoms of MS include optic neuritis, sensory loss, limb weakness, diplopia, gait ataxia, cognitive dysfunction, and loss of bladder control [3]. Although the underlying pathogenesis of MS remains obscure, evidence suggests the etiology of MS is probably multifactorial [3]. Research indicates that the peroxidation of biological molecules plays a significant role in the development of MS [4], which sheds light on the potential of antioxidants in MS management [5–9].

One of the most significant lipid‐soluble antioxidants is vitamin E, which includes two families of tocopherols (*α*, *β*, *γ*, and *δ* tocopherol) and tocotrienols (*α*, *β*, *γ*, and *δ* tocotrienols) [10, 11]. Among the various isoforms of vitamin E, *α*‐tocopherol was selected as the focus of this study because it is the predominant form maintained in human plasma and tissues due to the specific activity of the hepatic *α*‐tocopherol transfer protein, which preferentially retains *α*‐tocopherol and facilitates its biological availability [12, 13]. Furthermore, *α*‐tocopherol exhibits the highest biological potency among vitamin E isoforms and acts as a chain‐breaking antioxidant that protects cell membranes from oxidative lipid damage, a mechanism closely associated with the oxidative stress implicated in MS pathology [14, 15].

The possible association of serum vitamin E levels and MS pathogenesis was evaluated in previous research [16–20]. Despite the conflicting results in the conducted studies, there was no systematic review that determines the association of *α*‐tocopherol and pathogenesis of MS, which is addressed in the present study.

## 2. Method

This systematic review and meta‐analysis was executed in accordance with the recommendations established by the Preferred Reporting Items for Systematic Reviews and Meta‐Analyses (PRISMA) [21] and the Joanna Briggs Institute (JBI) manual for evidence synthesis. The study protocol has been registered in the International Prospective Register of Systematic Reviews (PROSPERO ID: CRD42023454925). Eligibility criteria for this systematic review are presented in Table 1.

**Table 1:** Eligibility criteria for this systematic review.

Inclusion criteria	Not inclusion or exclusion criteria
1. Investigating patients living with MS	1. Review articles, commentaries, case reports, case series
2. Presence of a healthy control group	2. Non‐English papers
3. Assessing the serum levels of *α*‐tocopherol in both groups	3. Animal studies
	4. Conference abstracts

Abbreviation: MS, multiple sclerosis.

### 2.1. Search Strategy

Four databases (Scopus, PubMed, Web of Science, and Embase) were independently searched for all the publications until March 23, 2025. The search strategy (Supporting Information 1) was developed by two independent authors (A.N. and H.A.) and peer reviewed by two other researchers (L.N. and S.S.). Additionally, the reference lists of the selected studies were reviewed to ensure that no relevant articles were overlooked. In order to comprehensively identify all pertinent publications, the automated notification feature of the PubMed database was utilized.

### 2.2. Study Selection and Risk of Bias (RoB) Assessments

After the removal of duplicates in EndNote software, the final library was imported into Rayyan [22]. The blind mode was applied. Four investigators (H.A., R.A., N.S., and S.P.L.) independently differentiated the eligible articles for this systematic review according to their titles/abstracts and full‐text. RoB was evaluated utilizing the JBI critical appraisal tools [23, 24]. Two of the authors (R.D. and H.A) carried out the RoB assessments. Discrepancies in the screening process and RoB assessments were first discussed to achieve consensus and unresolved conflicts and discrepancies were settled by a third author (S.H. or M.T.), whose decision was considered final.

### 2.3. Data Extraction

The required data were independently extracted by two of the authors (R.A. and H.A.) and checked by two other researchers (B.A. and N.S.), and discrepancies were settled by a third author (A.N. or S.H.). Information was gathered from each selected study, comprising the name of the first author, the year of publication, and the setting of the study. Additionally, participant characteristics such as the sample size, expanded disability status scale (EDSS), type of MS, age, sex, and diagnostic criteria were documented. Details regarding the serum *α*‐tocopherol in the study groups and assessment method, and the conclusions drawn from the study were also extracted.

### 2.4. Data Integration and Statistical Evaluation

Meta‐analysis was conducted using the comprehensive meta‐analysis software (CMA3) and considering the great level of heterogeneity, based on the *Q* statistics and *I* ^2^ index, a random‐effects model analysis was employed to calculate the aggregated effect size [25, 26]. The effect size was reported as standardized mean difference (SMD) in conjunction with 95% confidence intervals (CIs) and the threshold for statistical significance was set at *p* < 0.05. The prediction interval was computed using the pooled effect estimate ±1.96 × √(SE^2^ + *τ* ^2^), where SE is the standard error of the pooled effect and *τ* ^2^ represents the between‐study variance. Subgroup analyses were performed to identify the potential sources of heterogeneity, incorporating factors such as sample size, sex, and disease phase.

## 3. Results

### 3.1. Study Selection

**Figure 1:**
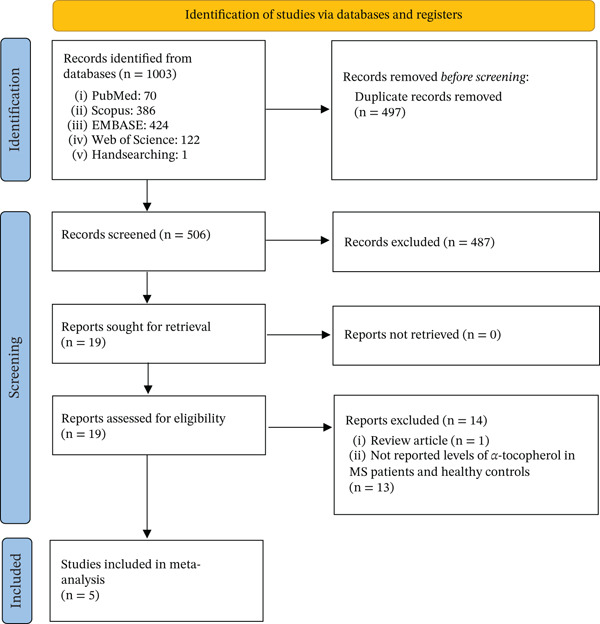
PRISMA flow diagram.

As depicted in Figure 1, PRISMA flow diagram, the initial search yielded 1003 records. After eliminating duplicate entries, 506 records underwent screening based on titles and abstracts. Subsequently, 19 papers remained and were subjected to full‐text evaluation. From these, 14 papers including one review article [27] and 13 studies that did not report serum levels of *α*‐tocopherol in MS patients and healthy controls [28–40] were excluded. Ultimately, five case‐control studies met the eligibility criteria [16–20].

### 3.2. Study Characteristics

Table 2 summarizes the demographic characteristics of the participants in the selected papers. Participant numbers ranged from 29 to 137 across studies. The studies were executed in the United States [20], the United Kingdom [17], Spain [18], Italy [19], and Türkiye [16].

**Table 2:** Characteristics of the included studies.

Study	Setting	Sample size		Age (MS/Control)		Female ratio (MS/Control)		EDSS	Type of MS	MS diagnostic criteria	*α*‐tocopherol assessment method, reported unit	Serum *α*‐tocopherol (crude data)		Comparison of MS and HC
		MS	HC	MS	HC	MS	HC					MS	HC	
Ghebremeskel et al. 1988 [17]	United Kingdom	18	11	25–42	24–54	83.3%	NR	5.5, 3.75, 4.75 ^∗^	RRMS (100%)	NR	HPLC, mgr/L	5.5 ± 0.41, 7.8 ± 0.35, 10.0 ± 0.54 ^∗^	6.5 ± 0.25	Higher in medium and high intake groups but not in unsupplemented MS group
Salemi et al. 2010 [19]	Italy	40	80	39.5	38.5	70%	70%	3.0 (1.0–6.0)	RRMS (70%)/SPMS (30%)	NR	HPLC and fluorimetry, mgr/L	12.0 (5.4, 26.5)	22.5 (1.9, 70.2)	Lower in MS patients
Jiménez‐Jiménez et al. 1998 [18]	Spain	36	32	31.9 ± 10.2	31.6 ± 8.9	66.6%	65.6%	NR	NR	Poser	HPLC, mmol/L	27.7 ± 6.5	32.6 ± 9.1	Lower in MS patients
Besler et al. 2002 [16]	Turkey	24	24	35.9 ± 7.3	36.8 ± 4.1	33.3%	33.3%	NR	SPMS	Poser	Spectrofluorometer, mmol/L	22.05 ± 2.05	26.20 ± 1.99	Lower in MS patients
Nehzat et al. 2024 [20]	Buffalo, United States	106	31	45.0 ± 10.6; 56.4 ± 6.39 ^∗∗^	46.2 ± 12.8	72%; 80%	64%	2.0 (1.5, 3.0); 5.0 (3.5, 6.5) _∗∗_	RRMS (67%)/PMS (33%)	McDonald 2010	HPLC, *μ*g/mL	13.5 ± 4.6; 13.9 ± 4.1 ^∗∗^	12.7 ± 4.89	No significant difference between HC, RRMS, and PMS

*Note:*  ^∗^ In low, medium, and high intake groups.  ^∗∗^ In relapsing‐remitting, and progressive forms.

Abbreviations: EDSS, expanded disability status scale; HCL, healthy control; HPLC, high performance liquid chromatography; MS, multiple sclerosis; NR, not reported; RRMS, relapsing‐remitting multiple sclerosis; SPMS, secondary progressive multiple sclerosis.

### 3.3. RoB assessment

The methodological quality of the included studies is presented in Table 3. These studies demonstrated methodological strength in key areas, encompassing case‐control comparability, standardized exposure measurement, and valid outcome assessment. The only possible source of bias in Jiménez‐Jiménez et al. 1998 [18], Salemi et al. 2010 [19], and Besler et al.′s [16] studies was an unclear exposure period. In addition, the lack of appropriate matching between cases and controls and the nonexclusion of supplemented participants were another possible source of bias in Nehzat et al.′s study [20]. Ghebremeskel et al.′s study [17] was identified as a study with high RoB considering the lack of appropriate matching and dealing with confounders. Age, sex, and receiving supplementation as the most common confounding factors were identified and addressed in the other included studies.

**Table 3:** Risk of bias assessment of the case‐control studies based on JBI critical appraisal tool.

Study	1	2	3	4	5	6	7	8	9	10	Total score
Salemi et al. 2010 [19]	Y	Y	Y	Y	Y	Y	Y	Y	UC	Y	9
Ghebremeskel et al. 1988 [17]	N	N	N	Y	Y	Y	N	Y	UC	Y	5
Jiménez‐Jiménez et al. 1998 [18]	Y	Y	Y	Y	Y	Y	Y	Y	UC	Y	9
Besler et al. 2002 [16]	Y	Y	Y	Y	Y	Y	Y	Y	UC	Y	9
Nehzat et al. 2024 [20]	Y	N	Y	Y	Y	N	N	Y	UC	Y	6

Abbreviations: N, no; N/A, not applicable; UC, unclear; Y, yes.

### 3.4. *α*‐Tocopherol Concentrations in MS and Healthy Control Groups

**Figure 2:**
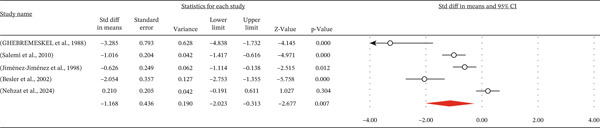
The forest plot of serum *α*‐tocopherol concentrations in patients with MS compared to healthy controls.

Besler et al.′s study [16] found that serum levels of antioxidant vitamins (ascorbic acid, *α*‐tocopherol, retinol and *β*‐carotene) were significantly lower in secondary progressive MS patients who first experience exacerbations compared to healthy controls. Their study also demonstrated increased lipid peroxidation in patients living with MS, indicating an increased oxidative stress burden. Researchers suggested further evaluation of the possible benefits of vitamin supplementation in prevention or treatment of MS. Jiménez‐Jiménez et al. 1998 [18] examined cerebrospinal fluid (CSF) and serum levels of *α*‐tocopherol in individuals suffering from MS, all during the disease exacerbation, and found that although serum levels were lower in MS individuals compared to healthy controls, CSF levels were not significantly different. This suggests that peripheral vitamin E levels may not directly reflect CNS oxidative stress. Additionally, the study found no link between *α*‐tocopherol concentrations and age, age at onset, and duration of the disease. Regarding the inactive phase of the disease, Nehzat et al. 2024 [20], a five‐year follow‐up assessment of retinol, tocopherols, and carotenoids in individuals with MS, found that *α*‐tocopherol levels were linked to EDSS, serum neurofilament light chains, and age. Salemi et al. 2010 [19] also reported significantly lower serum vitamin E levels in MS individuals during the phase of clinical inactivity compared to healthy controls; finally, Ghebremeskel et al.′s study [17] also found comparable levels of *α*‐tocopherol concentrations between unsupplemented MS patients and healthy controls. The results of this meta‐analysis, based on five case‐control studies [16–20] with a combined sample size of 389 participants, comprising 211 individuals diagnosed with MS and 178 healthy controls, have indicated that *α*‐tocopherol concentrations are significantly lower in MS patients than healthy controls (SMD = −1.17; 95% CI: −2.02, −0.31; *p* = 0.007; predictive values: −3.13, 0.79), despite considerable heterogeneity (*I* ^2^ = 91.5*%*, *p* < 0.001) (Figure 2). As illustrated in Table 4, subgroup analysis was performed to find the potential source of heterogeneity, and the results revealed no significant alterations. Leave‐one‐out sensitivity analyses, with excluding the Ghebremeskel et al.′s study [17], was not associated with a significant change in the observed heterogeneity (*I* ^2^ = 91.75*%*, *p* < 0.001); and the difference was still marginally significant (*p* value = 0.047).

**Table 4:** Subgroup analyses of comparison of serum *α*‐tocopherol concentrations in MS patients and healthy controls.

Group	Effect size (number)	SMD (95% CI)	*I* ^2^(%), *p* for heterogeneity
**Total**	5	−1.17 (−2.02, −0.31)	91.5%, < 0.001
***Sample size***			
> 68	2	−0.04 (−1.60, 0.80)	94.4%, < 0.001
≤ 68	3	−1.84 (−3.21, −0.47)	88.9%, < 0.001
***Sex***			
Female dominancy	4	−0.90 (−1.77, −0.04)	90.3%, < 0.001
Male dominancy	1	−2.05 (−2.75, −1.35)	—
***Disease phase***			
Attack	2	−1.32 (−2.72, 0.08)	90.7, 0.001
Inactive	3	−1.12 (−2.40, 0.16)	93.50, < 0.001

Abbreviation: SMD, standardized mean difference.

## 4. Discussion

This meta‐analysis assessed the link between serum concentration of *α*‐tocopherol and MS pathogenesis. Despite considerable heterogeneity, the quantitative synthesis in this study suggested significantly lower concentrations of *α*‐tocopherol in MS patients. Substantial observed heterogeneity may be attributed to geographic differences, different MS diagnostic criteria and phenotypes, timing of measurement, and varying dietary patterns among study populations. In addition to the heterogeneity, considering the concerns regarding potential publication bias and small number of included studies, overall certainty of the evidence in the present meta‐analysis is very low. In addition, the lack of temporal assessments restricts conclusions about the causal role of *α*‐tocopherol in MS progression and further prospective cohort studies are recommended.

Considering the antioxidant effects, the role of vitamin E in the immune system is suggested [41, 42]. In addition, its role in brain health and its neuroprotective effects are still under investigation [43, 44], and Langemann et al. [45] have shown that vitamin E levels decreased in the plaques of demyelination of MS brains. Despite the suggested association of *α*‐tocopherol and MS pathogenesis, higher vitamin E intake was not found to be associated with a reduced risk of MS [33]. Also, evidence on the potential of vitamin E‐enriched diets such as the Mediterranean diet is conflicting [46, 47], which should be further evaluated.

Numerous studies demonstrated the beneficial suppressive effect of vitamin E on systemic peroxidation in people living with MS [32, 37, 39]. In a study of an animal model of MS, Haikuo Xue et al. [48] reported amelioration of experimental autoimmune encephalomyelitis (EAE) with *α*‐tocopherol supplementation, which suppresses the proliferation of T cells and the Th1 response. Animal studies have also shown the potential for myelin regeneration, emphasizing the role of vitamin E as a complementary therapeutic agent for MS [49, 50]. The main meta‐analysis in this study suggested a significant association, which suggested benefits of vitamin E supplementation and consumption of plant‐based oils (such as olive oil), nuts, seeds, dark leafy green vegetables, and avocados in MS.

This meta‐analysis employed a rigorous methodological framework to ensure the comprehensive inclusion of the studies. However, this study also has considerable restrictions. Excluding non‐English articles created language bias and the substantial heterogeneity among selected studies may restrict the reliability of pooled estimates. Additionally, this meta‐analysis included only five case‐control studies, which may increase the risk of publication bias and limit the generalizability of the findings.

## 5. Conclusion

Serum *α*‐tocopherol concentrations are suggested to be significantly lower in individuals suffering from MS compared to healthy controls, which shed light on the possible association between MS pathogenesis and serum *α*‐tocopherol concentrations. Given the significance of oxidative stress in MS pathogenesis, further well‐designed research utilizing longitudinal design is warranted.

## Author Contributions

H.A., B.A., N.S., R.A., S.P.L., S.H., R.D., and A.N.: systematic search, study selection, data extraction, preparing the figures, formal analysis, drafting the manuscript, risk of bias assessment, and drafting the manuscript. A.N., H.A., S.S., M.T., and L.N.: conceptualization and supervision. H.A. and B.A. have contributed to the work equally and should be regarded as cofirst authors.

## Funding

This study was supported by the Tabriz University of Medical Sciences, 10.13039/501100004366, grant number: 72313.

## Disclosure

All authors approved the final version for submission.

## Ethics Statement

The ethics committee of Tabriz University of Medical Sciences reviewed and approved the study protocol (ethics code: IR.TBZMED.FMD.REC.1404.143).

## Consent

The authors have nothing to report.

## Conflicts of Interest

The authors declare no conflicts of interest.

## Supporting information


**Supporting Information** Additional supporting information can be found online in the Supporting Information section. File S1: The details of search strategies.

## Data Availability

All data generated or analyzed during this study are included in this published article.

## References

[1] Shi M. , Liu Y. , Gong Q. , and Xu X. , Multiple Sclerosis: An Overview of Epidemiology, Risk Factors, and Serological Biomarkers, Acta Neurologica Scandinavica. (2024) 2024, no. 1, 7372789, 10.1155/2024/7372789.

[2] Walton C. , King R. , Rechtman L. , Kaye W. , Leray E. , Marrie R. A. , Robertson N. , la Rocca N. , Uitdehaag B. , van der Mei I. , Wallin M. , Helme A. , Angood Napier C. , Rijke N. , and Baneke P. , Rising Prevalence of Multiple Sclerosis Worldwide: Insights From the Atlas of MS, third edition, Multiple Sclerosis Journal. (2020) 26, no. 14, 1816–1821, 10.1177/1352458520970841, 33174475. 33174475 PMC7720355

[3] Papiri G. , D’Andreamatteo G. , Cacchiò G. , Alia S. , Silvestrini M. , Paci C. , Luzzi S. , and Vignini A. , Multiple Sclerosis: Inflammatory and Neuroglial Aspects, Current Issues in Molecular Biology. (2023) 45, no. 2, 1443–1470, 10.3390/cimb45020094, 36826039. 36826039 PMC9954863

[4] Vasić M. , Topić A. , Marković B. , Milinković N. , and Dinčić E. , Oxidative Stress-Related Risk of the Multiple Sclerosis Development, Journal Of Medical Biochemistry. (2023) 42, no. 1, 1–8, 10.5937/jomb0-37546, 36819128. 36819128 PMC9920994

[5] Salekzamani S. , Pakkhesal S. , Babaei M. , Mirzaaghazadeh E. , Mosaddeghi-Heris R. , Talebi M. , Sanaie S. , and Naseri A. , Coenzyme Q10 Supplementation in Multiple Sclerosis; a Systematic Review, Multiple Sclerosis and Related Disorders. (2025) 93, 106212, 10.1016/j.msard.2024.106212, 39667129. 39667129

[6] Jiménez-Jiménez F. J. , Alonso-Navarro H. , Salgado-Cámara P. , García-Martín E. , and Agúndez J. A. G. , Antioxidant Therapies in the Treatment of Multiple Sclerosis, Biomolecules. (2024) 14, no. 10, 10.3390/biom14101266, 39456199. PMC11506420 39456199

[7] Morsali S. , Sabahi Z. , Kakaei J. , Hakimzadeh Z. , Hamidi S. , Gholipour-Khalili E. , Sanaie S. , Talebi M. , and Naseri A. , Clinical Efficacy and Safety of Melatonin Supplementation in Multiple Sclerosis: A Systematic Review, Inflammopharmacology. (2023) 31, no. 5, 2213–2220, 10.1007/s10787-023-01271-4, 37429996. 37429996

[8] Naseri A. , Sanaie S. , Rahnemayan S. , Mosaddeghi-Heris R. , Talebi M. , and Talebi M. , The Effects of Probiotic Supplementation on Level of Disability, Depressive Symptoms, and Cognitive Outcomes in Relapsing-Remitting Multiple Sclerosis Patients: A Randomized Double-Blind Placebo-Controlled Trial, Pharmaceutical Sciences. (2025) 31, no. 3, 322–329, 10.34172/PS.025.41013.

[9] Abbasi H. , Shakouri F. , Mosaddeghi-Heris R. , Gholipour-Khalili E. , Jahanshahlou F. , Sanaie S. , Naseri A. , and Talebi M. , Mediterranean-Like Diets in Multiple Sclerosis: A Systematic Review, Revue Neurologique. (2024) 180, no. 10, 1021–1030, 10.1016/j.neurol.2023.07.017, 39492055. 39492055

[10] Villalón-García I. , Álvarez-Córdoba M. , Povea-Cabello S. , Talaverón-Rey M. , Villanueva-Paz M. , Luzón-Hidalgo R. , Suárez-Rivero J. M. , Suárez-Carrillo A. , Munuera-Cabeza M. , Salas J. J. , Falcón-Moya R. , Rodríguez-Moreno A. , Armengol J. A. , and Sánchez-Alcázar J. A. , Vitamin E Prevents Lipid Peroxidation and Iron Accumulation in PLA2G6-Associated Neurodegeneration, Neurobiology of Disease. (2022) 165, 105649, 10.1016/j.nbd.2022.105649, 35122944. 35122944

[11] Kaye A. D. , Thomassen A. S. , Mashaw S. A. , MacDonald E. M. , Waguespack A. , Hickey L. , Singh A. , Gungor D. , Kallurkar A. , Kaye A. M. , Shekoohi S. , and Varrassi G. , Vitamin E (*α*-Tocopherol): Emerging Clinical Role and Adverse Risks of Supplementation in Adults, Cureus. (2025) 17, no. 2, e78679, 10.7759/cureus.78679, 40065887. 40065887 PMC11891505

[12] Brigelius-Flohé R. and Traber M. G. , Vitamin E: Function and Metabolism, FASEB Journal. (1999) 13, no. 10, 1145–1155, 10.1096/fasebj.13.10.1145. 10385606

[13] Traber M. G. and Atkinson J. , Vitamin E, Antioxidant and Nothing More, Free Radical Biology & Medicine. (2007) 43, no. 1, 4–15, 10.1016/j.freeradbiomed.2007.03.024, 17561088. 17561088 PMC2040110

[14] Traber M. G. and Stevens J. F. , Vitamins C and E: Beneficial Effects From a Mechanistic Perspective, Free Radical Biology & Medicine. (2011) 51, no. 5, 1000–1013, 10.1016/j.freeradbiomed.2011.05.017, 21664268. 21664268 PMC3156342

[15] Galli F. , Azzi A. , Birringer M. , Cook-Mills J. M. , Eggersdorfer M. , Frank J. , Cruciani G. , Lorkowski S. , and Özer N. K. , Vitamin E: Emerging Aspects and New Directions, Free Radical Biology & Medicine. (2017) 102, 16–36, 10.1016/j.freeradbiomed.2016.09.017, 27816611. 27816611

[16] Besler H. T. , Çomoğlu S. , and OkÇu Z. , Serum Levels of Antioxidant Vitamins and Lipid Peroxidation in Multiple Sclerosis, Nutritional Neuroscience. (2002) 5, no. 3, 215–220, 10.1080/10284150290029205, 12041878. 12041878

[17] Ghebremeskel K. , Williams G. , Harbige L. S. , and Forti A. D. , Plasma Retinol and *α*-Tocopherol Concentrations in Supplemented and Unsupplemented Multiple Sclerosis Patients, Journal of Clinical Biochemistry and Nutrition. (1988) 5, no. 1, 81–85, 10.3164/jcbn.5.81.

[18] Jiménez-Jiménez F. J. , de Bustos F. , Molina J. A. , de Andrés C. , Gasalla T. , Ortí-Pareja M. , Zurdo M. , Porta J. , Castellano-Millán F. , Arenas J. , and de Salamanca R. E. , Cerebrospinal Fluid Levels of Alpha-Tocopherol in Patients With Multiple Sclerosis, Neuroscience Letters. (1998) 249, no. 1, 65–67, 10.1016/S0304-3940(98)00370-X, 9672390. 9672390

[19] Salemi G. , Gueli M. C. , Vitale F. , Battaglieri F. , Guglielmini E. , Ragonese P. , Trentacosti A. , Massenti M. , Savettieri G. , and Bono A. , Blood Lipids, Homocysteine, Stress Factors, and Vitamins in Clinically Stable Multiple Sclerosis Patients, Lipids in Health and Disease. (2010) 9, no. 1, 19–23, 10.1186/1476-511X-9-19. 20163740 PMC2834681

[20] Nehzat N. , Browne R. W. , Ghazal D. , Tamaño-Blanco M. , Jakimovski D. , Weinstock-Guttman B. , Zivadinov R. , and Ramanathan M. , Exploratory 5-Year Follow-Up Study of Retinol, Tocopherols, and Carotenoids in Multiple Sclerosis, Multiple Sclerosis and Related Disorders. (2024) 81, 105143, 10.1016/j.msard.2023.105143, 38039941. 38039941

[21] Page M. J. , McKenzie J. E. , Bossuyt P. M. , Boutron I. , Hoffmann T. C. , Mulrow C. D. , Shamseer L. , Tetzlaff J. M. , Akl E. A. , Brennan S. E. , Chou R. , Glanville J. , Grimshaw J. M. , Hróbjartsson A. , Lalu M. M. , Li T. , Loder E. W. , Mayo-Wilson E. , McDonald S. , McGuinness L. A. , Stewart L. A. , Thomas J. , Tricco A. C. , Welch V. A. , Whiting P. , and Moher D. , The PRISMA 2020 Statement: An Updated Guideline for Reporting Systematic Reviews, International Journal of Surgery. (2021) 88, 105906, 10.1016/j.ijsu.2021.105906, 33789826. 33789826

[22] Ouzzani M. , Hammady H. , Fedorowicz Z. , and Elmagarmid A. , Rayyan—A Web and Mobile App for Systematic Reviews, Systematic Reviews. (2016) 5, no. 1, 1–10, 10.1186/s13643-016-0384-4. 27919275 PMC5139140

[23] Tufanaru C. , Munn Z. , Aromataris E. , Campbell J. , and Hopp L. , Systematic Reviews of Effectiveness, Joanna Briggs Institute Reviewer′s Manual, 2017, The Joanna Briggs Institute Adelaide, 3–10.

[24] Moola S. , Munn Z. , Tufanaru C. , Aromataris E. , Sears K. , Sfetcu R. , Currie M. , Qureshi R. , Mattis P. , Lisy K. M. , and Mu P. F. , Chapter 7: Systematic Reviews of Etiology and Risk, Joanna Briggs Institute Reviewer’s Manual. (2017) 5, 217–269.

[25] Cochran W. G. , The Combination of Estimates From Different Experiments, Biometrics. (1954) 10, no. 1, 101–129, 10.2307/3001666.

[26] Higgins J. P. and Thompson S. G. , Quantifying Heterogeneity in a Meta-Analysis, Statistics in Medicine. (2002) 21, no. 11, 1539–1558, 10.1002/sim.1186, 12111919. 12111919

[27] Allan Butterfield D. , Castegna A. , Drake J. , Scapagnini G. , and Calabrese V. , Vitamin E and Neurodegenerative Disorders Associated With Oxidative Stress, Nutritional Neuroscience. (2002) 5, no. 4, 229–239, 10.1080/10284150290028954. 12168685

[28] Warren T. , Multiple Sclerosis and Infants Fed on Diets Deficient in Vitamin A or in Selenium and Vitamin E, Medical Hypotheses. (1982) 8, no. 5, 443–454, 10.1016/0306-9877(82)90003-2. 7109983

[29] Ghabaee M. , Jabedari B. , Al-E-Eshagh N. , Ghaffarpour M. , and Asadi F. , Serum and Cerebrospinal Fluid Antioxidant Activity and Lipid Peroxidation in Guillain–Barre Syndrome and Multiple Sclerosis Patients, International Journal of Neuroscience. (2010) 120, no. 4, 301–304, 10.3109/00207451003695690, 20374079. 20374079

[30] Aristotelous P. , Stefanakis M. , Pantzaris M. , Pattichis C. S. , Calder P. C. , Patrikios I. S. , Sakkas G. K. , and Giannaki C. D. , The Effects of Specific Omega-3 and Omega-6 Polyunsaturated Fatty Acids and Antioxidant Vitamins on Gait and Functional Capacity Parameters in Patients With Relapsing-Remitting Multiple Sclerosis, Nutrients. (2021) 13, no. 10, 10.3390/nu13103661, 34684661. PMC8540949 34684661

[31] Atuk Kahraman T. , Yılmaz M. , Yetkin M. F. , and Mirza M. , The Nutritional Status of Relapsing-Remitting Multiple Sclerosis (RRMS) Patients Compared to That of Healthy People: A Turkish Hospital-Based Study, Nutritional Neuroscience. (2022) 25, no. 11, 2279–2287, 10.1080/1028415X.2021.1956253, 34311682. 34311682

[32] Guan J.-Z. , Guan W.-P. , and Maeda T. , Vitamin E Administration Erases an Enhanced Oxidation in Multiple Sclerosis, Canadian Journal of Physiology and Pharmacology. (2018) 96, no. 11, 1181–1183, 10.1139/cjpp-2018-0246, 30092167. 30092167

[33] Zhang S. , Hernan M. , Olek M. , Spiegelman D. , Willett W. , and Ascherio A. , Intakes of Carotenoids, Vitamin C, and Vitamin E and MS Risk Among Two Large Cohorts of Women, Neurology. (2001) 57, no. 1, 75–80, 10.1212/WNL.57.1.75, 11445631. 11445631

[34] Dowd G. C. , Massive Dosage of Alpha-Tocopherol in Alleviation of Multiple Sclerosis, Annals of the New York Academy of Sciences. (1949) 52, no. 3, 422–424, 10.1111/j.1749-6632.1949.tb55308.x.

[35] Hadžović-Džuvo A. , Lepara O. , Valjevac A. , Avdagić N. , Hasić S. , Kiseljaković E. , Ibragić S. , and Alajbegović A. , Serum Total Antioxidant Capacity in Patients With Multiple Sclerosis, Bosnian Journal of Basic Medical Sciences. (2017) 11, no. 1, 33–36, 10.17305/bjbms.2011.2620, 21342139. PMC4362562 21342139

[36] Hunter M. I. S. , Lao M. S. , Burtles S. S. , and Davidson D. L. , Erythrocyte Antioxidant Enzymes in Multiple Sclerosis and the Effect of Hyperbaric Oxygen, Neurochemical Research. (1984) 9, no. 4, 507–516, 10.1007/BF00964377, 6462325. 6462325

[37] Besler H. T. and Çomogˇlu S. , Lipoprotein Oxidation, Plasma Total Antioxidant Capacity and Homocysteine Level in Patients With Multiple Sclerosis, Nutritional Neuroscience. (2003) 6, no. 3, 189–196, 10.1080/1028415031000115945, 12793524. 12793524

[38] Karg E. , Klivenyi P. , Bencsik K. , Turi S. , and Vecsei L. , Alpha-Tocopherol and NADPH in the Erythrocytes and Plasma of Multiple Sclerosis Patients, European Neurology. (2003) 50, no. 4, 215–219, 10.1159/000073862, 14634265. 14634265

[39] Røsjø E. , Myhr K.-M. , Løken-Amsrud K. I. , Bakke S. J. , Beiske A. G. , Bjerve K. S. , Hovdal H. , Lilleås F. , Midgard R. , Pedersen T. , Šaltytė Benth J. , Torkildsen Ø. , Wergeland S. , Michelsen A. E. , Aukrust P. , Ueland T. , and Holmøy T. , Increasing Serum Levels of Vitamin A, D and E Are Associated With Alterations of Different Inflammation Markers in Patients With Multiple Sclerosis, Journal of Neuroimmunology. (2014) 271, no. 1-2, 60–65, 10.1016/j.jneuroim.2014.03.014, 24713402. 24713402

[40] Løken-Amsrud K. I. , Myhr K.-M. , Bakke S. J. , Beiske A. G. , Bjerve K. S. , Bjørnarå B. T. , Hovdal H. , Lilleås F. , Midgard R. , Pedersen T. , Benth J. Š. , Torkildsen Ø. , Wergeland S. , and Holmøy T. , Alpha-Tocopherol and MRI Outcomes in Multiple Sclerosis–Association and Prediction, PLoS One. (2013) 8, no. 1, e54417, 10.1371/journal.pone.0054417, 23349882. 23349882 PMC3551804

[41] Rezaieyazdi Z. , Sahebari M. , Saadati N. , and Khodashahi M. , Vitamin E and Autoimmune Diseases: A Narrative Review, Reviews in Clinical Medicine. (2018) 5, no. 2, 42–48.

[42] Rezaeimanesh N. , Jahromi S. R. , Moghadasi A. N. , Ghorbani Z. , Eskandarieh S. , and Sahraian M. A. , Is a Higher Dietary Intake of Vitamin E Related to Lower Odds of Neuromyelitis Optica Spectrum Disorder?, Multiple Sclerosis and Related Disorders. (2020) 37, 101560, 10.1016/j.msard.2019.11.035.

[43] Boccardi V. , Baroni M. , Mangialasche F. , and Mecocci P. , Vitamin E Family: Role in the Pathogenesis and Treatment of Alzheimer′s Disease, Alzheimer′s & Dementia: Translational Research & Clinical Interventions. (2016) 2, no. 3, 182–191, 10.1016/j.trci.2016.08.002, 29067305. PMC5651353 29067305

[44] Lakhan R. , Sharma M. , Batra K. , and Beatty F. B. , The Role of Vitamin E in Slowing Down Mild Cognitive Impairment: A Narrative Review, Healthcare. (2021) 9, no. 11, 10.3390/healthcare9111573. PMC8625211 34828619

[45] Langemann H. , Kabiersch A. , and Newcombe J. , Measurement of Low-Molecular Weight Antioxidants, Uric Acid, Tyrosine and Tryptophan in Plaques and White Matter From Patients With Multiple Sclerosis, European Neurology. (1992) 32, no. 5, 248–252, 1521544. 1521544 10.1159/000116835

[46] Shakouri F. , Lotfi M. , Rostami A. , Talebi M. , Sanaie S. , and Naseri A. , Association Between Mediterranean Diet and Development of Multiple Sclerosis: A Systematic Review and Meta-Analysis, Brain and Behavior: A Cognitive Neuroscience Perspective. (2026) 16, no. 2, e71205, 10.1002/brb3.71205, 41612786. PMC12856230 41612786

[47] Ratti S. , Eke H. , Cantarutti A. , Bonn S. E. , Adami H. O. , Ye W. , Ponzano M. , Grotta A. , and Trolle Lagerros Y. , Mediterranean Diet and Risk of Multiple Sclerosis: A Prospective Cohort Study, Multiple Sclerosis Journal. (2026) 32, no. 1, 61–68, 10.1177/13524585251396408, 41399205. 41399205 PMC12756519

[48] Xue H. , Ren H. , Zhang L. , Sun X. , Wang W. , Zhang S. , Zhao J. , and Ming L. , Alpha-Tocopherol Ameliorates Experimental Autoimmune Encephalomyelitis Through the Regulation of Th1 Cells, Iranian Journal of Basic Medical Sciences. (2016) 19, no. 5, 561–566, 27403263. 27403263 PMC4923477

[49] Goudarzvand M. , Javan M. , Mirnajafi-Zadeh J. , Mozafari S. , and Tiraihi T. , Vitamins E and D3 Attenuate Demyelination and Potentiate Remyelination Processes of Hippocampal Formation of Rats Following Local Injection of Ethidium Bromide, Cellular and Molecular Neurobiology. (2010) 30, no. 2, 289–299, 10.1007/s10571-009-9451-x, 19768531. 19768531 PMC11498802

[50] Khosravi-Largani M. , Pourvali-Talatappeh P. , Rousta A. M. , Karimi-Kivi M. , Noroozi E. , Mahjoob A. , Asaadi Y. , Shahmohammadi A. , Sadeghi S. , Shakeri S. , Ghiyasvand K. , and Tavakoli-Yaraki M. , A Review on Potential Roles of Vitamins in Incidence, Progression, and Improvement of Multiple Sclerosis, eNeurologicalSci. (2018) 10, 37–44, 10.1016/j.ensci.2018.01.007, 29736427. 29736427 PMC5934114

