# Supplementary material for: The Association Between Serum α‐Tocopherol and Pathogenesis of Multiple Sclerosis: A Systematic Review and Meta‐Analysis of Case‐Control Studies
Source: Biomed Res Int. 2026 Jul 8;2026:2590873. doi: 10.1155/bmri/2590873 (PMC13346587; doi:10.1155/bmri/2590873)
Supplement: Supplementary file 1 — Supporting Information Additional supporting information can be found online in the Supporting Information section. File S1: The details of search strategies. [file BMRI-2026-2590873-s001.docx]

**Supplementary Material 1 – The details of search strategies.**

**PubMed:**

("vitamin e"[All Fields] OR ("Tocopherols"[MeSH Terms] OR "Tocopherols"[All Fields] OR "tocopherol"[All Fields] OR "tocopheryl"[All Fields]) OR ("tocotrienols"[MeSH Terms] OR "tocotrienols"[All Fields] OR "tocotrienol"[All Fields]) OR "Tocopherols"[MeSH Terms] OR "vitamin e"[MeSH Terms]) AND "Multiple Sclerosis"[All Fields]

**Scopus:**

TITLE-ABS-KEY ( ( "vitamin E"  OR  tocopherol  OR  tocotrienol )  AND  ( "Multiple Sclerosis" ) )

**Embase:**

('vitamin e'/exp OR 'vitamin e' OR 'tocopherol'/exp OR tocopherol OR 'tocotrienol'/exp OR tocotrienol) AND ('multiple sclerosis'/exp OR 'multiple sclerosis')

**Web of Science:**

("vitamin E" OR Tocopherol OR Tocotrienol) AND ("Multiple Sclerosis") (All Fields)
